# Geographic Variation in Strabismus Pattern Among Pediatric Age Group in Lebanon: A Single-Centre Five-Year Observational Study

**DOI:** 10.7759/cureus.15957

**Published:** 2021-06-27

**Authors:** Hani Chanbour, Ayman Bsat, Wassef Chanbour, Carol Cherfan

**Affiliations:** 1 Ophthalmology, Faculty of Medicine, Lebanese University, Beirut, LBN; 2 Ophthalmology, Lebanese University, Beirut, LBN; 3 Ophthalmology, Beth Israel Deaconess Medical Center (BIDMC), Boston, USA; 4 Ophthalmology, Beirut Eye and ENT Specialist Hospital, Beirut, LBN

**Keywords:** strabismus, pediatrics, esotropia, exotropia, amblyopia, refractive error

## Abstract

Background: Strabismus means ocular misalignment. It is also one of the most prevalent types of amblyopia and the leading cause of pediatric visual impairment.

Objective: This study aims to determine the frequency of different types of strabismus and the associated refractive errors and amblyopia in patients younger than 16 years of age. This study also aims to compare the age at presentation and gender between the geographic locations and between different strabismus types.

Materials and methods: A cross-sectional retrospective study was done using the archives of Beirut Eye and ENT specialist hospital between January 2014 and December 2018. Lebanese pediatric patients aged <16 years having strabismus were included in this study.

Results: There was a total of 787 pediatric patients with strabismus, 62.6% of cases had esotropia (ET) and 30.2% exotropia (XT), with ET/XT: 2.07/1. Mt Lebanon had the highest number of cases, whereas Nabatieh was the only governorate with reversed ET/XT ratio. Most patients were diagnosed at the age of 1-5 years, with ET being the most common diagnosis, while XT was mostly found in patients aged 11-15. Hyperopia was the most common (55.4%) refractive error detected in our cohort of strabismus patients, followed by myopia and simple astigmatism. Amblyopia was found in 18.9% of cases, where Nabatieh had the highest count.

Conclusion: Strabismus pattern was investigated for the first time across Lebanon to shed the light on the crucial role of early ophthalmologic examination, to detect early refractive error and strabismus, and to prevent amblyopia.

## Introduction

The strabismus is derived from the Greek word strabismus “to squint” and it means ocular misalignment. It is the most prevalent binocular vision disorder that also affects stereopsis and depth perception. Worldwide prevalence of strabismus was reported to range between 2% and 5% in school-aged children [1]. Other global population studies like the Multi-Ethnic Pediatric Eye Disease Study (MEPEDS) [2,3], the Baltimore Pediatric Eye Disease Study (BPEDS) and the Strabismus, Amblyopia, and Refractive Error in Singaporean Children Study (STARS) [4] reported 0.8% to 3.5% prevalence of strabismus in preschool and school-aged children.

Strabismus varies between different countries and ethnic backgrounds; it is reported to be more common among Whites in Europe than those living in the United States. In addition, strabismus was more common in white Americans compared to African Americans [5].

Furthermore, the ratio of esotropia (ET) to exotropia (XT) varies in different parts of the world, ET (inward deviation of the eye) had a 75% prevalence in the US while XT (outward deviation) was the most prevalent in Japanese children [5].

Many environmental and genetic factors were implicated in the development of strabismus. Few examples include low birth weight, preterm birth, congenital abnormalities, a large head circumference [1], astigmatism [6], cerebral palsy, intraventricular bleeding, maternal cigarette smoking during pregnancy, parental age [7], and the presence of retinopathy of prematurity [8], among others.

Strabismus is one of the most common causes of amblyopia, the leading cause of pediatric visual impairment. It is defined as a unilateral or bilateral reduction of best-corrected visual acuity [9]. It develops during the critical period of visual system maturation when there is visual deprivation or abnormal binocular interaction. Often, children with unilateral amblyopia have a higher risk of age-related macular degeneration and bilateral visual impairment later in life. Early detection and treatment of this condition, especially at a younger age, can lead to improved visual acuity [10].

To our knowledge, no reports have been published discussing the strabismus patterns in the Lebanese population, and due to the high frequency of consanguinity in many areas of the country, we suppose that the frequency of strabismus types may vary depending on the location.

This study aims to determine the frequency of different types of strabismus, refractive errors and amblyopia in patients younger than 16 years of age, diagnosed with strabismus examined at Beirut eye and ENT specialist hospital between January 2014 and December 2018, in addition to their geographic distribution in Lebanon.

This study also aims to identify the different refractive errors and amblyopia in each strabismus type, and to compare the age at presentation and gender, previous use of spectacles, and other associated ophthalmic pathologies, between the geographic locations and between different strabismus type.

## Materials and methods

Study design

This retrospective cross-sectional study was conducted using data from the archives of Beirut Eye and ENT specialist hospital, where all charts created for the patients examined January 2014 and December 2018 in the pediatric ophthalmology department were reviewed.

Study population

Inclusion criteria: Lebanese nationality, age less than 16 years, and the patient has had to have strabismus identified after undergoing complete ocular examination including cover test, Krimsky test, measuring corrected distance visual acuity (CDVA), manifest refraction, cycloplegic refraction, slit lamp, and fundus evaluations. Exclusion criteria: all non-Lebanese patients were excluded from the study.

Definitions

• Myopia is defined as a spherical equivalent refractive error of at least -0.75 D in one or both eyes.

• Hypermetropia is defined as a spherical equivalent refractive error of at least +2.00 D or more in one or both eyes.

• Astigmatism is defined (as cylinder powers ≥ 0.50 DC) if one or both eyes were astigmatic.

• Manifest strabismus is defined as constant or intermittent tropia of any magnitude at distance or near fixation.

• Accommodative ET was defined as >2.00 D of hyperopia, uncorrected ET of > 10Δ for distance or near, and corrected ET of ≤ 10Δ for distance and near sight with the use of full cycloplegic hyperopic correction.

• Finally, unilateral amblyopia was defined as a two-line difference in CDVA between both eyes and one eye CDVA is worse than 20/32.

Data collection

A special form was used to input data on the age, gender, type and amount of refractory error, the presence of strabismus and its type, the treatment administered, and the presence of associated conditions with strabismus in addition to anterior and posterior eye abnormalities. The approval was obtained from the Institutional Review Board (IRB) at the Beirut Eye and ENT Specialist Hospital, with adherence to the Declaration of Helsinki, IRB approval number 2021-05.

Statistical analysis

SPSS software (version 21, SPP, Inc., Chicago, IL, USA) was used to determine the prevalence frequency of each identified strabismus cases and types. In addition, the t-test and chi-square test were used to determine if a statistically significant relationship existed between the age at diagnosis and the type of strabismus and refractive errors present. For inferential statistics when exploring relationships between variables and recurrence rate in each group, an independent t-test was used for continuous variables (age) and a chi-square test (Fisher's Exact Test) was used for categorical variables (type of strabismus, type of refractive error and the governorate). P-value was considered significant if less than 0.05.

## Results

Prevalence of strabismus

**Figure 1:**
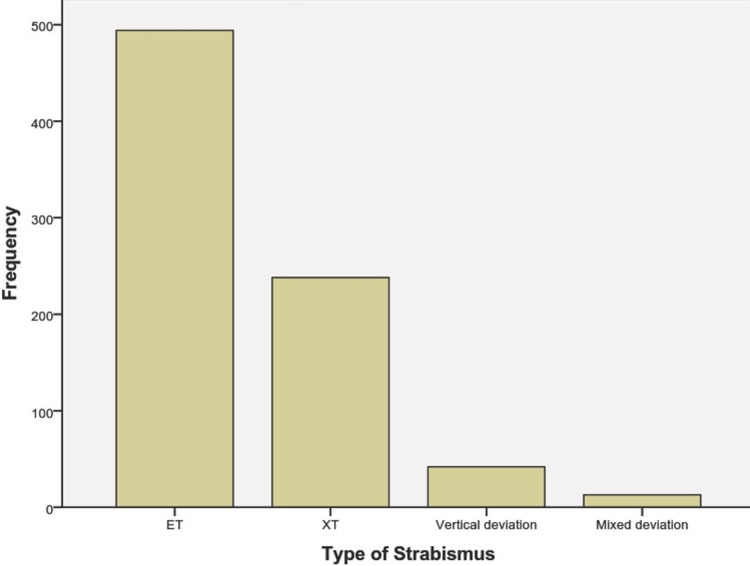
The number of each type of strabismus and the number of patients diagnosed with each type of strabismus ET: esotropia XT: exotropia

**Figure 2:**
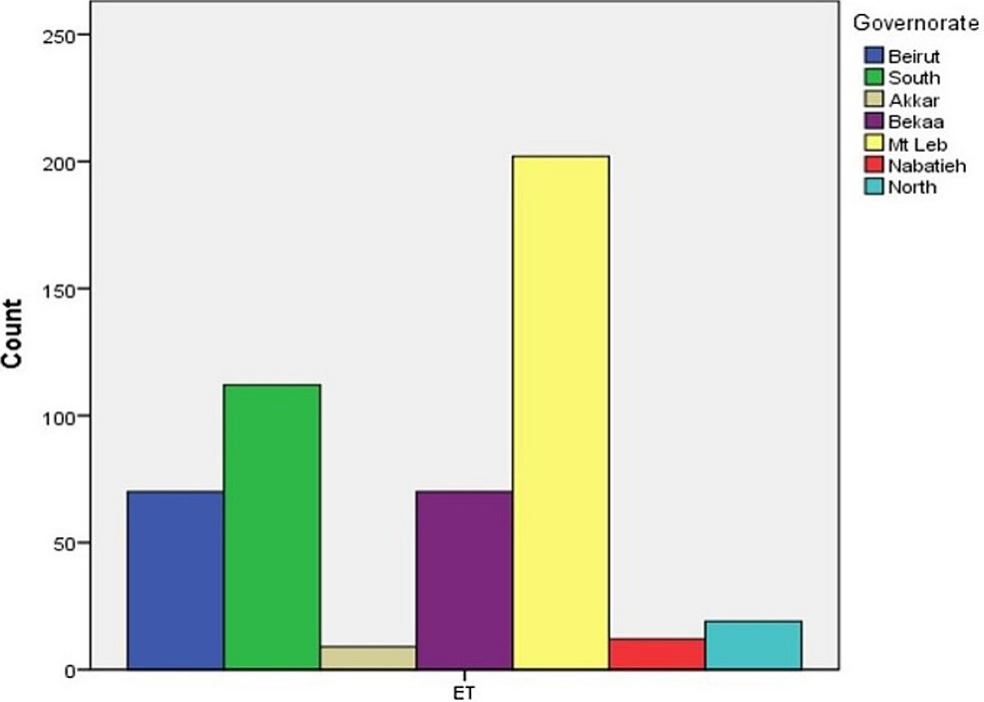
Geographic distribution of esotropia patients ET: esotropia

**Figure 3:**
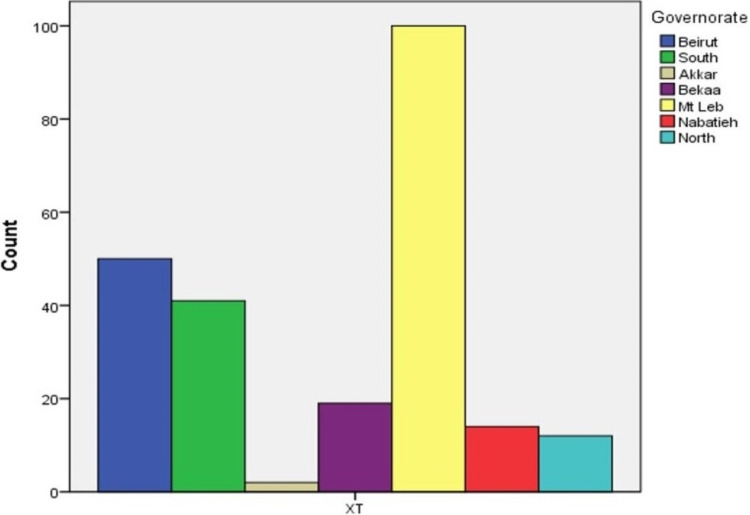
Geographic distribution of exotropia patients XT: exotropia

Between January 2014 and December 2018, a total of 787 pediatric patients were diagnosed with strabismus at our institution. Among this cohort, ET is the most common type of strabismus, accounting for 62.6% of all cases, followed by XT at 30.2%. Of those diagnosed with ET, 31.1% were refractive type, 14.6% were partially accommodative, and 30.5% were undefined (Figures 1-3).

We had 403 (52%) female cases compared to 384 (48%) male cases of strabismus. The distribution of the types of strabismus based on gender was non-significant (p = 0.052).

The association between refractive errors and strabismus

For the sake of simplicity, we combined myopia and myopic astigmatism patients and hyperopia and hyperopic astigmatism patients. We found that hyperopia was the most common (55.4%) refractive error detected in our cohort of strabismus patients, followed by myopia and simple astigmatism. 373 (75.5%) of the 494 patients diagnosed with ET had hyperopia, 45 (9.1%) had hyperopia and astigmatism and only 10 (2.0%) had myopia. On the other hand, 46 (19.3%) of the 238 patients diagnosed with XT had hyperopia, 29 (12.2%) had myopia and astigmatism and 40 (16.8%) had myopia only. A total of 147 (18.9%) patients had amblyopia. Of those, 57 (38.9%) patients had a refractive error in addition to strabismus, 31 (21.5%) patients had a partially accommodative error and 33 (22.8%) patients had an undefined error. There was no statistical significance with regards to the strabismus type and the presence of amblyopia.

Subtypes of strabismus

**Figure 4:**
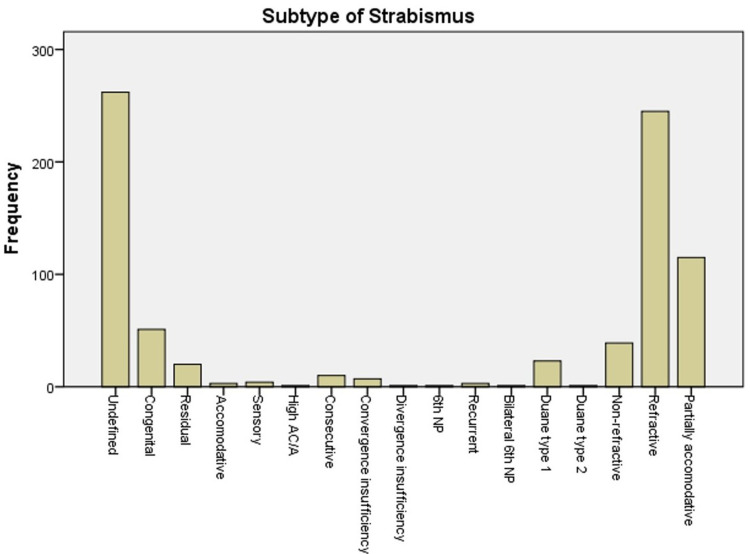
Number of each type of strabismus

Fourth nerve palsy was reported in 2.2% of the patients presenting with strabismus, whereas sixth nerve palsy was seen in only one child with ET. Similarly, concomitance in strabismus was reported in 94% of our cohort and only 6% of our cohort showing non-comitant strabismus (Figure 4).

Geographic and age distribution of strabismus cases 

**Figure 5:**
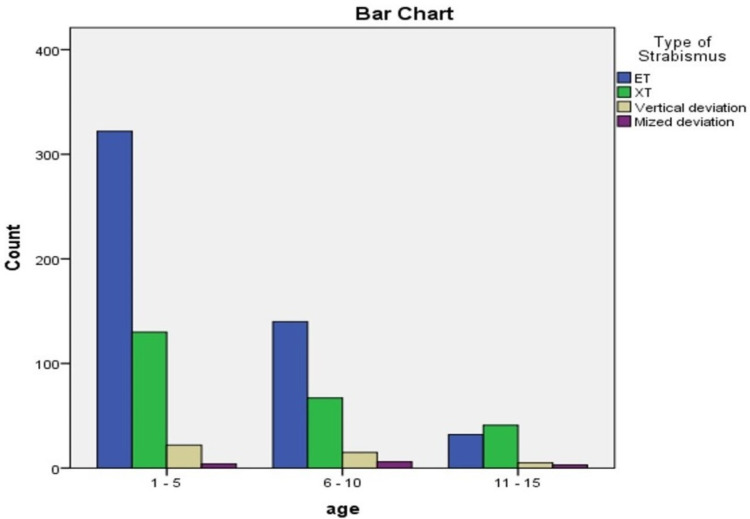
Frequency of strabismus types in each age range ET: esotropia XT: exotropia

With regards to demographic data, Mt Lebanon had the highest number of strabismus patients presenting to the hospital. Interestingly, ET had a higher prevalence than XT in every governorate but the Nabatieh governorate. Most of our cohort was between ages 1 and 5 years when they received their diagnosis of strabismus, with age 3 being the most common age at diagnosis (Figure 5).

ET was more common than XT in all ages, except the 11-15 age group, where XT was more diagnosed than ET. Chi-square analysis of the type of strabismus and age at diagnosis yields a p-value < 0.001.

Management of strabismus

**Figure 6:**
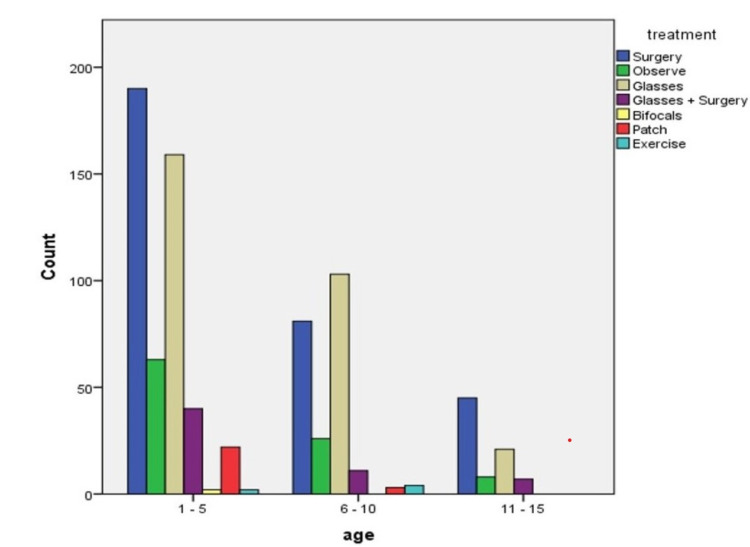
Treatment of strabismus in each age group

The most common treatment modalities were prescription glasses and surgery, conservative treatment and prescription glasses in all age groups. Eyeglasses correction alone was the most common treatment, followed by observation and surgery at ages 1-10 (Figure 6).

## Discussion

In this study, we investigated the proportion of strabismus pattern across Lebanon for the first time, in patients younger than 16 years of age diagnosed between January 2014 and December 2018.

Out of 787 strabismus cases, we found 494 (62.8%) cases of ET, 238 (30.2%) of XT, 42 (5.3%) cases of Vertical Deviation, and 32 (1.7%) cases of Mixed Deviation. Our study showed that ET in the present study was more common than XT, with a ratio of 2.07/1. ET/XT ratios between 2.6/1 and 4.9/1 in children have been reported previously in Sweden, Turkey, India, and Scotland [1].

All the Lebanese governorates were found to have more ET patients than XT, except for Nabatieh where the ratio was 1/1.1. The difference between different geographic locations and ethnicities in the same country was reported in the literature, especially among children. Robaei and colleagues studied the strabismus patterns in Australia among 1,739 children aged 6-7 years, the ratio of ET/XT among the complete population was 1.8:1, whereas the east Asian population in Australia had more XT cases with a ratio of 1:1.6 [6]. In addition, a study involved 17,018 children aged 4-5, found a total ratio of 1:1.2, whereas white British children had 1.3:1, and Pakistani children had 1:75 [1]. This highlights the difference in the same country, according to different ethnicities and genes involved [3]. Accommodative ET is linked to moderate to high hyperopia so the decreased incidence of hyperopia would lower the prevalence of accommodative ET, thus increasing the XT/ET ratio, which can explain the different results found in these studies as there are lower hyperopia rates in east Asian population [3,5]. It is well understood nowadays that refractive errors, including hyperopia, are influenced by both biological (nature) as well as environmental (nurture) sources. The hyperopic shift occurs between the ages of 40 and 60. Thus, younger populations may have a smaller iris and so the higher the prevalence of hyperopia and thus less more ET. Certain genes may predispose individuals to develop hyperopia and these genes may become more prevalent in certain societies due to consanguinity, or geographic isolation [1].

Mt Lebanon comprised the highest number of strabismus cases referred to the hospital (323 cases, 41%) whereas Akkar had the smallest number of strabismus cases (14 cases, 1.7%). This small number can be attributed to the large geographic distance between Akkar and Beirut and the socioeconomic factors that may impede referrals to tertiary care centers. This can lead to the lack of hospital visits and basic access to ophthalmologic examination, which may cause underdiagnosis and under-reporting of eye pathologies.

Regarding age distribution, most patients were initially diagnosed with strabismus at ages 1 to 5, followed by the 6 to 10 years' age group and only 10% of strabismus patients were first diagnosed at ages 11-16 years. Moreover, in our study, ET was more common between 1 and 10 years of age, and XT was more common in 11-16 years of age. This was comparable to other studies: A study found that ET was detected between 1 and 4 years of age, whereas older children had predominance of XT [11,12]. Furthermore, one study had found more ET in children aged 3-10 years, and XT in 11-16 years of children. They also showed a higher prevalence of strabismus among children aged 3-10 years of age (0.38%) compared to children of 11-16 years of age (0.22%) [13].

Our cohort featured an almost equal number of male and female patients. ET was diagnosed more in boys while XT was diagnosed more in girls, but there was no statistical significance. This result was comparable to other studies. One study found 46.8% prevalence in boys, and 53.2% in girls [14], whereas a study found 52.3% prevalence of strabismus in boys compared with 47.7% in girls. Again, in these studies, the results were not statistically significant [15]. In addition, female predominance in XT is a well-elucidated finding. Another study reported that XT was twice as prevalent in females as males, but there were no significant differences in clinical findings [16].

There were 740 (94%) cases of comitant strabismus, and 47 (6%) cases of non-comitant strabismus. This was comparable to other studies. The frequency of concomitant strabismus was 76 (87.4%) in pediatric age group in a tertiary eye care hospital in Pakistan [1].

Among non-comitant strabismus cases, 23 (2.9%) were attributed to Duane type 1, and 1 (0.1%) case of Duane type 2. 2.2% of cases was attributed to fourth nerve palsy, and there was only one case of sixth nerve palsy. One study found that the most common cause of non-comitant strabismus was fourth nerve palsy (36%), followed by the sixth (33%), the third (22%), and multiple nerve palsies (9%). It is thought that congenital factors are responsible for fourth nerve palsy, but the cause is undetermined for the sixth nerve palsy [17]. A study concluded that a new onset sixth nerve palsy in children can be benign in 13% of cases, hence why a full physical exam and a thorough history is recommended, with brain MRI, to rule out the original cause [1].

The number of Amblyopia cases was exceptionally high in the Nabatieh governorate, which may be due to a lack of proper strabismus screening programs and a lack of regular eye check-ups. One study reported a 53% of amblyopia among strabismus cases; this was due to the lack of awareness among the patient’s parents, of the adverse outcome of untreated strabismus [1]. It was also assumed in the past that the strabismus brings good luck to the family, part of the parents’ refusal of surgery for their children. In addition to that, girls would not seek surgery until they reach marriageable age, by that time, there is loss of stereopsis, and amblyopia would develop [14]. However, a study on 9172 Asian and non-Hispanic white children found demonstrated a 1.81% amblyopia rate [3]. A meta-analysis involving 73 studies concluded a prevalence estimate of amblyopia to be 1.75% (95% CI: 1.62-1.88) [18].

Hyperopia was the most common refractive error in ET and XT; it was present in 85.5% of cases of esotropic patients and 33.3% of exotropic patients. On the other hand, 80% of myopia and 65.2% of astigmatism cases were observed in patients with XT. It seems that hyperopia was associated with ET while myopia and astigmatism were associated with XT. MEPEDS and BPEDS concluded that hyperopia of +3.00 D or more was the main predictor of ET, which explains our results. In addition, there was a linear relationship between the prevalence of ET and the spherical equivalent refractive error. The odds ratio of having ET is 122 times greater in hyperopic children of 5.00 D or more compared with children with 1.00 D or less. Astigmatism of 2.50 D or more was the main predictor of XT, increasing the risk sixfold. Also, there is a linear relationship between astigmatism and XT [1]. MEPEDS also concluded that strabismus was associated with both spherical anisometropia (odds ratio of 6.2 for ET and 3.52 for XT), and cylindrical anisometropia (odds ratio 3.02 for ET and 3.99 for XT) [18].

Limitations of the study

There are several limitations to this study; the most significant is not having enough heterogeneity in the study population. Despite being a national referral center for eye pathology, certain governorates, particularly the rural North, South, and Beqaa regions in the country were under-represented while the urban and metropolitan regions were over-represented. In addition, we did not control for the socioeconomic status of our cohort. We suspect that our cohort does not accurately encompass the Lebanese population, particularly those living far away from the capital, Beirut, and coming from a lower socioeconomic background. This is only exacerbated by the fact that the local Lebanese population is a relatively small one to begin with. Thus, we are not entirely confident in the generalizability of our findings, given that most of our cohort consisted of urban dwellers belonging to middle and high socioeconomic status. Further, inter-disciplinary collaboration is needed with public health institutions, schools and primary health clinics, with a special emphasis on rural areas in Akkar, Bekaa, and the southern governorates to institute robust eye screening programs for children and be able to better characterize the prevalence of strabismus in Lebanon.

## Conclusions

In our study, we investigated the prevalence of different strabismus patterns across Lebanon for the first time, in patients younger than 16 years of age, examined at Beirut eye and ENT specialist hospital between January 2014 and December 2018. There were 787 cases of strabismus, with ET:XT ratio of 2.07:1, with comparable results in the populations elsewhere. No difference between males and females was seen. Strabismus was more commonly detected between 1 and 5 years of age, common in younger patients. Hyperopia was associated with ES, whereas myopia and astigmatism were related to XT. Mt Lebanon had the highest number of strabismus patients in our hospital, while Nabatieh had the largest proportion of amblyopia This may be due to the difference in socioeconomic status and access to nearby hospitals for ophthalmologic examinations.
